# In Situ Growth of Enamel-like Apatite Coating for Marble Protection

**DOI:** 10.3390/ma18040880

**Published:** 2025-02-17

**Authors:** Yihang Zhou, Wenfei Li, Yue Wang, Kai Wang

**Affiliations:** 1 Key Laboratory of Archaeomaterials and Conservation, Ministry of Education, Institute for Cultural Heritage and History of Science & Technology, University of Science and Technology Beijing, Beijing 100083, China; zhouyihang@ustb.edu.cn; 2 College of Chemistry and Molecular Engineering, Peking University, Beijing 100871, China; 3 College of Humanities and Law, Interdisciplinary Research Center for Science and Technology for Cultural Heritage, Beijing University of Chemical Technology, Beijing 100029, China; 4 The Institute for Archaeology, Beijing Union University, Beijing 100191, China

**Keywords:** marble conservation, artificial enamel, hydroxyapatite, enamel-like coating

## Abstract

Outdoor stone relics, including inscriptions, statues, temple grottoes, etc., are continuously subjected to natural weathering and air pollutants. Those made of marbles and other carbonate rocks are particularly vulnerable to acid rains, which can be protected by acid-resistant coatings. A novel method to prepare enamel-like hydroxyapatite coating on marble surfaces is presented in this paper and analyzed using optical microscopy, a scanning electronic microscope, grazing incident X-ray diffraction, and nano-indentation. The described coating is composed of tightly arranged hydroxyapatite nanorods, perpendicular to the marble substrate, with a thickness of 3–5 μm. Not only does the coating exhibit high acid resistance, it also has considerably higher elastic modulus and hardness compared to that synthesized by the well-known diammonium phosphate (DAP) method owing to the wellarranged microstructure. Consequently, the enamel-like hydroxyapatite coating would probably be more effective and durable for marble protection than the existing calcium phosphate coating.

## 1. Introduction

Stone relics, such as sculptures, monuments, inscriptions, and temple grottoes, often serve as tangible links to the past, providing insights into ancient civilizations, cultural practices, and historical events. They offer valuable information about the craftsmanship, beliefs, and traditions of the societies that created them. Moreover, many stone relics showcase exceptional artistic skill and aesthetic beauty. They represent important examples of ancient and classical art, contributing to our understanding of artistic techniques, styles, and cultural expressions from different periods and regions. Unfortunately, outdoor relics are constantly exposed to environmental threats, such as weathering, air pollution, and natural disasters, as well as from human activities. Therefore, conservation efforts typically aim to protect stone relics from these risks.

Categorizing stone relics by material type (e.g., marble, limestone, granite) is important for conservation practices, as different materials have unique properties and require specific preservation approaches. Marble and limestone are widely used in historic buildings and monumental constructions, such as the Taj Mahal in India [1] and Ancient Mayan structures [2]. These calcareous materials present unique conservation challenges when compared to other stone relics. This is largely due to marble’s specific chemical properties originating from carbonates and their susceptibility to acidic substances, such as acidic air pollutants (NO_X_, SO_2_), and CO_2_ in water. Acidic air pollutants have continuously caused the deterioration and blackening of historical monuments and buildings, especially those made of carbonates [3,4,5]. The microwear or surface erosion of the surfaces can be measured by an elastomeric tactile sensor to provide topographic information [6]. The weathering of marble and limestone not only leads to the impaired surface appearances [7,8], but it can also cause salt crystallization and sugaring of the stone [9,10]. Coating is a feasible and simple way to separate these valuable heritages from acid rains and other contaminants.

Acrylates, such as Paraloid B72, copolymer of methyl acrylate, and ethyl methacrylate, are the most common materials for coating artefact purpose, which has been applied in Longmen Grottoes and the dome of Rome [11]. Alkoxysilane based materials, such as TEOS, with good permeability and aging resistance, are also commonly used in stone conservation [12]. Despite the high effectiveness in silicate stones, they have unsatisfactory compatibility with carbonate stones [13]. Back in the 1980s, calcium oxalate coatings associated with metabolic activity of lichens and fungi were found to protect carbonate rocks [14]. Since then, attempts have been made by using oxalic acid or ammonium oxalate to converse carbonate to oxalate coatings on marble and limestone [15,16,17,18]. Hydroxyapatite (HAP) is the most stable species of calcium phosphates when pH > 4, with a solubility product constant Ksp much lower than both calcium carbonate and oxalate, and good compatibility with carbonates, which makes it suitable for conservation purposes [19]. The well-known diammonium phosphate (DAP) method was established by Sassoni E. in 2011 and optimized later, which effectively converses calcium carbonate to phosphates with acid resistance, including HAP and octacalcium phosphate (OCP) [20,21,22]. Naidu found that an addition of minor calcium source, such as CaCl_2_, would increase the coverage of phosphate coating on marbles, and high concentrations of DAP would lead to cracking [23]. Sassoni also improved the DAP method by introducing ethanol, or isopropanol (IPA), which effectively reduces the pores and micro fissures in the phosphate coating [24]. However, the above two modifications both led to the formation of OCP, whose stability is secondary to HAP. Xu and Li found that surfactants, such as cetyltrimethylammonium bromide (CTAB), help to improve acid resistance by reducing surface area and pore sizes [25]. By comparing various DAP methods and the diammonium oxalate method in terms of acid resistance, Naidu concluded that, although calcium oxalate coating is superior in the short term, the DAP method using 1 M DAP + 1 mM CaCl_2_ solution performs better in the long run by reducing the erosion rate by approximately 40% [26]. Recently, nano aluminum phosphate has been used to facilitate the formation of HAP coating and improve its integrity [27].

**Figure 1:**
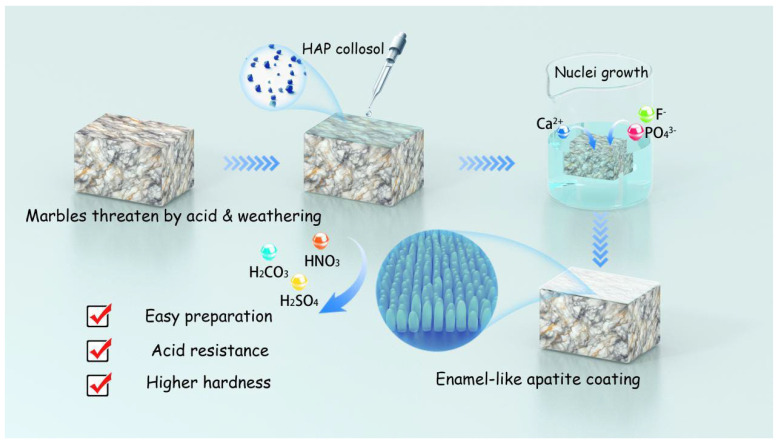
Scheme depicting the synthesis process of enamel-like apatite coating on marble.

When it comes to hydroxyapatite, enamel is supposed to be the most outstanding natural material composed of it. It is the outermost layer of tooth, with extraordinary toughness, stiffness, and acid resistance mainly owing to its unique aggregates of HAP nano rods. A variety of methods have been established to repair enamel for dental purpose and synthesize enamel-like novel materials [28,29,30,31,32]. In the current goal of coating marbles, very few efforts have been made to modulate the crystal morphology and arrangement. Presumably, an enamel-like HAP coating with compact nanorods perpendicular to the substrate would have better mechanical properties and acid resistance. After considering various artificial enamel synthesis techniques, we modified the one developed by Onuma K. and Iijima M. [33] and established a more suitable approach for marble protection (depicted in Figure 1). Specifically, HAP collosol containing countless crystal nuclei is applied to the marble surface. Subsequently, HAP crystal nuclei grow in a solution containing calcium, phosphate, and fluoride ions, which are close to, but not able to reach the upper limits of three-dimensional nucleation. Owing to the growth of HAP nano rods and geometrical selection [34,35] for each crystal, here we acquire an enamel-like HAP coating constituted by nanorods perpendicular to the marble surface. In this study, we made attempts to eliminate micro cracks in the coating, and characterize the morphology, acid resistance, and mechanical properties of the enamel-like HAP coating in comparison to the ones prepared by DAP methods.

## 2. Materials and Methods

### 2.1. Materials

The marble sample containing dolomite and calcite was cut into 20 × 20 × 17 mm in size and one face was polished by a 1000 mesh carborundum disk, if not otherwise stated, or a 400 or 180 mesh. NH_4_H_2_PO_4_, K_2_HPO_4_ and (NH_4_)_2_HPO_4_ were purchased from Sinopharm Chemical Reagent, Beijing, China. Anhydrous sodium acetate, citric acid monohydrate, and CaCl_2_ were purchased from Xilong Scientific, Guangdong, China. Acetic acid, ethanol, isopropanol, ammonia water, and Ca(NO_3_)_2_·7H_2_O were purchased from Tongguang Fine Chemical. NaF was purchased from Liyi Fine Chemical, Beijing, China. All reagents were analytical reagents and used directly.

### 2.2. Preparation of HAP Collosol

The HAP collosol was prepared according to Wang K. [36]. Specifically, 17.7 g Ca(NO_3_)_2_·7H_2_O and 10.5 g citric acid monohydrate were dissolved in 60 mL deionized water, followed by an addition of 15.0 mL ammonia water (d = 0.91), which is labeled as calcium solution. The phosphate solution was prepared by dissolving 5.2 g ammonium dihydrogen phosphate in 60 mL deionized water with an addition of 10.5 mL ammonia water. Under vigorous stirring, the phosphate solution was rapidly poured into the calcium solution, stirring continued at room temperature for 2 h, and then the solution was heated in boiling water bath for 20 min to obtain undesalted HAP collosol. The collosol was then dialyzed in deionized water at 80 °C until NH^4+^ concentration fell below 1 mg/mL, which was determined by Nessler’s reagent. After dialysis, the HAP collosol was concentrated into 50 mL at 90 °C and stored at 4 °C before use.

### 2.3. In Situ Growth of Enamel-like Apatite Coating

The growth of enamel-like apatite coating on marbles mainly involves two steps, as shown in Figure 1. Firstly, the as-prepared 1.0 wt.% HAP collosol is applied in a ratio of 10–100 μg per 1 cm^2^ marble surface by brushing. After the collosol is naturally dried, the marble is immersed in a pH 6.30 ± 0.05 0.05 mol/L sodium acetate buffer solution containing 4.00 mmol/L CaCl_2_, 4.00 mmol/L K_2_HPO_4_, and 0.526 mmol/L NaF for 2–48 h (24 h if not otherwise stated), which is called “mother solution”. The ratio of solution volume to the marble’s surface is 5 mL/cm^2^, if not otherwise stated, or 10 mL/cm^2^. Different conditions are listed in Table 1 and discussed along with the observation results. After the growth of coating, the samples were dried naturally in a lab environment at 20 ± 5 °C and 40 ± 10% RH.

### 2.4. Phosphate Coatings Prepared by DAP Methods

For the purpose of comparison, two DAP methods were adopted to prepare phosphate coatings on calcite marbles (due to the poor reactivity of DAP with dolomite). The first one was prepared according to reference [13] by immersing a marble sample in a solution containing 1 mmol/L CaCl_2_ and 1 mol/L diammonium phosphate for 48 h. The other was prepared according to reference [14] by immersing a marble sample in a solution containing 0.1 mmol/L CaCl_2_ and 0.1 mol/L diammonium phosphate and 10 *v*/*v*% isopropanol for 48 h. After the precipitation of coatings, the samples were dried naturally in lab environment at 20 ± 5 °C and 40 ± 10% RH.

### 2.5. Scanning Electron Microscopy with Energy Dispersive Spectroscopy (SEM-EDS)

During the optimization of the synthesis method, the microstructures of the coatings were directly observed by SEM (Hitachi TM3030, Tokyo, Japan) under low vacuum mode. Higher resolution SEM images were photographed by a Tescan Mira4 (Prague, Czech Republic) under high vacuum mode after the samples were coated with Au for better conductivity. The chemical composition was tested by Bruker Quantax70 (Bremen, Germany) EDS.

### 2.6. Grazing Incidence X-Ray Diffraction (GIXRD)

GIXRD (PANalytical X’Pert Powder X, Almelo, The Netherlands) was used to analyze the crystal phase of coating in situ. The XRD analysis covered the range of 2θ from 10° to 70° and used a Cu Kα 1 radiation source.

### 2.7. Polarized Microscopy

The sample was first cut into an approximately 1–2 mm slice, polished on one side with 1000 mesh and then stuck on a glass slide. The section was then polished to approximately 30 μm. Thin sections of the coating were observed under a Nikon ECLIPSE LV100N POL (Tokyo, Japan).

### 2.8. Acid Resistance Measurement

Each sample was coated with Paraloid B72, except for an area of 12 × 12 mm. They were immersed in solutions of 20 mL nictric acid with an initial pH of 4.0 and monitored by a METTLER TOLEDO SevenEasy pH meter (Greifensee, Switzerland) with a planar electrode at a distance of 1 mm from the marbles’ surface.

### 2.9. Nano Indentation

Mechanical properties were evaluated by a KLA G200 nano indenter (Milpitas, CA, USA) equipped with Berkovich indenter. The pressed depth was 1.0 μm, load resolution was 50 nN, and displacement resolution was 0.01 nm. At least 6 valid tests were conducted for each set of sample.

## 3. Results and Discussion

### 3.1. Optimization Against Cracking

**Figure 2:**
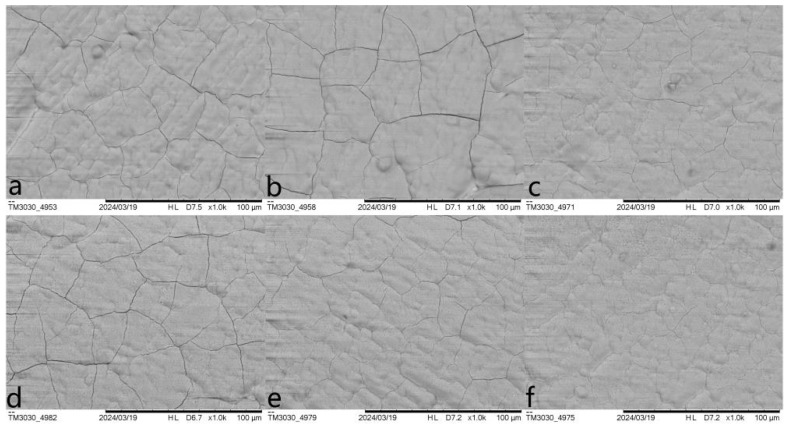
SEM images of enamel-like apatite coating corresponding to different conditions listed in Table 1: (**a**) entry 2; (**b**) entry 4; (**c**) entry 5; (**d**) entry 7; (**e**) entry 8; (**f**) entry 9.

**Figure 3:**
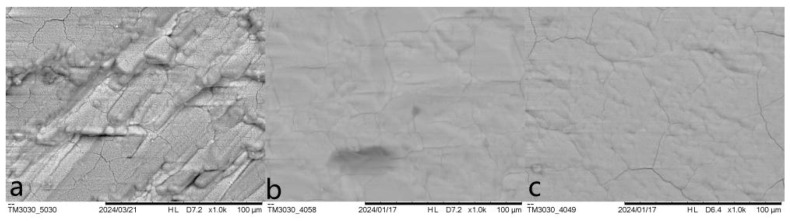
SEM images of enamel-like apatite coating on marbles polished by (**a**) 180 mesh; (**b**) 400 mesh; (**c**) 1000 mesh carborundum disk.

**Figure 4:**
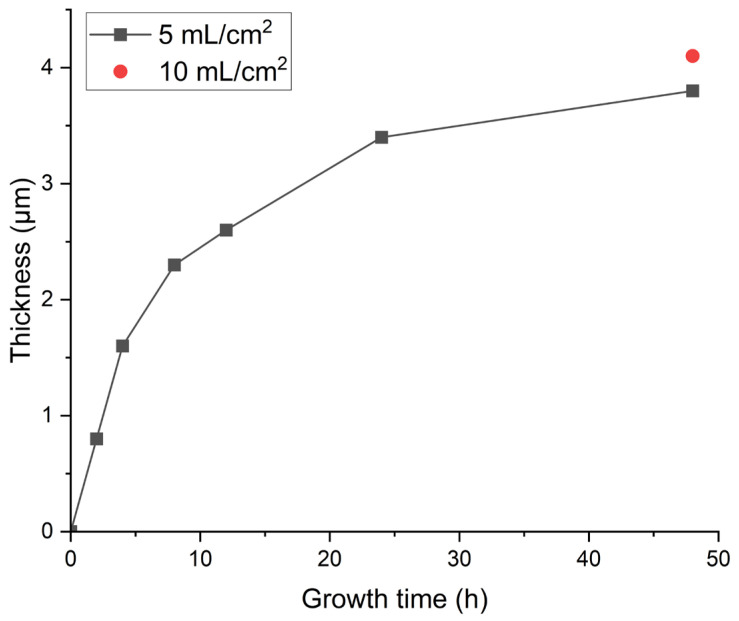
Thickness of the enamel-like coating (5 mL/cm^2^ means 5 mL mother solution per 1 cm^2^ marble’s surface).

Micro crack has always been a tricky problem when preparing phosphate coating on marbles, and enamel-like apatite coating is not an exception. The coating prepared by the basic method (labeled as Enamel method) shows an overall satisfying coverage of the marble surface, but had micro cracks of different levels. A variety of strategies have been attempted to minimize these micro cracks, including supplying additives in the mother solution, changing thickness (by the growth of time), limiting the amount of HAP collosol, etc. The specific conditions and observation results are summarized in Table 1. Although none of the variants or conditions led to a coating free of micro cracks, the amount of HAP collosol applied on the marble surface is found to be the key factor to the attachment of apatite coating on the marble’s surface and the formation of micro cracks. The coating would be opaque and flaking when an excess of HAP collosol is applied. By limiting HAP collosol addition, a transparent and well-attached coating can be acquired and still retained after scraping with nails. Micro cracks were effectively reduced when HAP collosol addition was controlled to the level of 10 μg/cm^2^ (shown in Figure 2c, compared to a and b). This observation suggests that the stress formed within the HAP collosol is more significant than the cracks in the coating. As both alcohol and CTAB were found to be helpful for reducing micro cracks in other phosphate coatings [24,25], they were added into the mother solution with a concentration of 10 *v*/*v*% and 0.01 mol/L, respectively, and turn out to be basically ineffective in further relieving cracking (Figure 2d,e). However, CTAB is effective in preventing phosphate solutions from growing microorganisms and might have other effects on the coating’s properties. The coating prepared by Enamel-CTAB method (entry 8) was further studied in the following sessions. Finally, surface etching may help to improve surface conjunction between HAP collosol and the surface of marble. As DAP is well accepted in the protection of marble, a pretreatment of DAP (entry 9 and Figure 2f) with proper application of HAP collosol were found to be optimal for preparing a coating with the least micro cracks, which is labeled as Enamel-DAP method. A short-time pretreatment of DAP would not lead to crystal precipitation as no coating was formed without the application of HAP collosol as nuclei (Table 1, entry 10). The Enamel-DAP method can be applied on marbles with different surface roughness (polished by 180, 400, 1000 mesh carborundum disks), as shown in Figure 3. The thickness can be controlled by the growth of time from 0 to 4 μm, and 5 mL mother solution is adequate for 1 cm^2^ of the marble’s surface (shown in Figure 4).

### 3.2. Morphology and Phase Characterization

**Figure 5:**
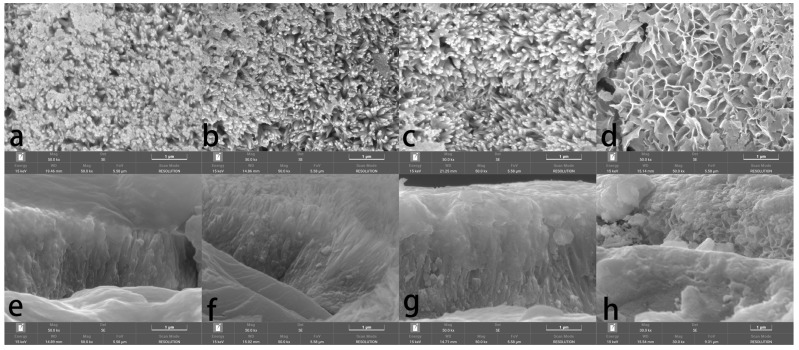
SEM images of enamel-like apatite coatings: top view (**a**) and side view (**e**) of the one prepared by Enamel method, top view (**b**) and side view (**f**) of the one prepared by Enamel-DAP method, top view (**c**) and side view (**g**) of the one prepared by Enamel-CTAB method, and top view (**d**) and side view (**h**) of the one prepared by DAP method.

The basic Enamel method and two modified methods, Enamel-DAP and Enamel-CTAB, were further characterized by SEM under a higher resolution in comparison with the DAP method. All the coatings in Figure 5a–c,e–g show enamel-like features, i.e., compact nano rods mostly perpendicular to the marble’s surface. The spatial orientations of the crystals were not strictly parallel since the marble’s surface is not perfectly smooth. The coating prepared by the DAP method is mainly composed of flakes in random orientation and it is less compact than the enamel-like coatings, which possibly compromise the mechanical properties and acid resistance. The chemical composition of the enamel-like coatings is shown in Table 2. The Ca/P ratios of the coatings are between 1.75 and 1.79, which is slightly higher than the theoretical value of HAP 1.67 and suggests a slight substitution of PO_4_^3−^ by CO_3_^2−^. The P/F ratios are between 3.80 and 3.92, which suggests that the OH^−^ in HAP is mostly substituted by F^−^. The coatings prepared by the three methods are overall the same in terms of micro morphology and chemical composition.

**Figure 6:**
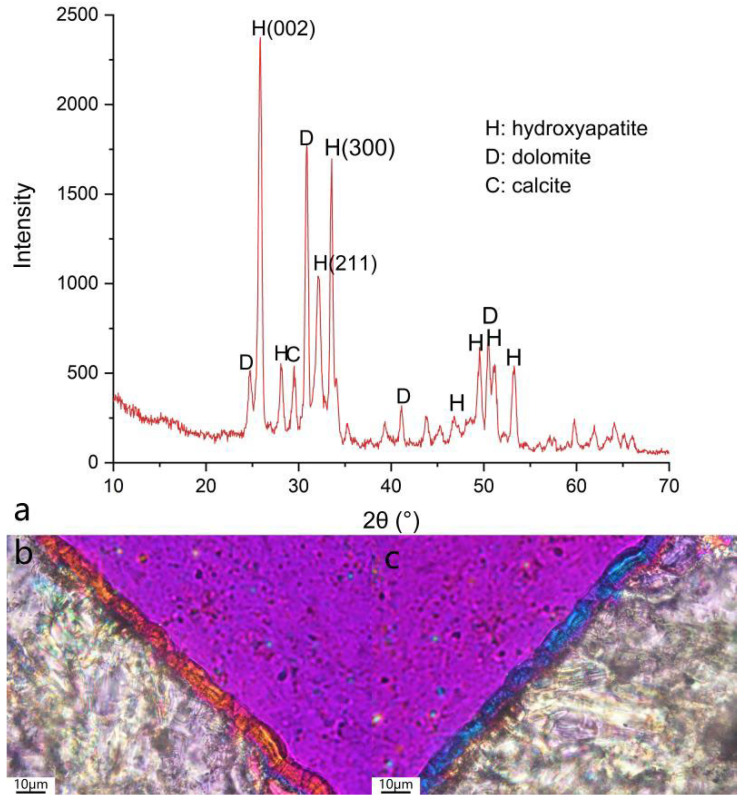
Phase characterization: (**a**) GIXRD spectrum of the enamel-like apatite coating, (**b**,**c**) thin section of the coating under cross-polarized microscope with gypsum compensator showing the well-oriented feature by interference colors.

The crystals in the enamel-like coating are further examined by GIXRD and polarized microscopy, as shown in Figure 6. Apart from the diffraction peaks of dolomite and calcite of the marble substrate, peaks at 25.8°, 32.2°, and 33.6°, clearly suggest the formation of HAP crystals with no other phosphate phase [37]. More intuitively, these crystals with negative elongation together formed a coating exhibiting parallel extinction (shown in Figure 6b), which again reveals the nature of these well-oriented HAP nano rods.

### 3.3. Acid Resistance

**Figure 7:**
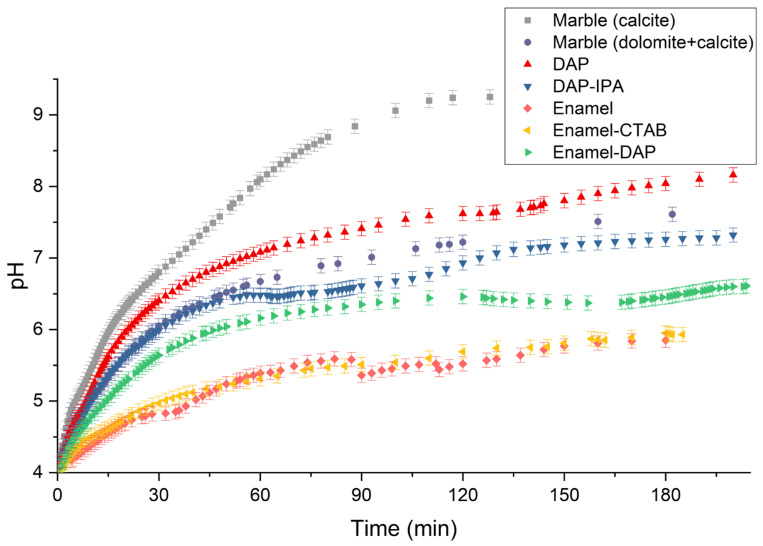
pH variations of the coatings prepared by different methods in pH4.0 nitric acid solution.

The acid resistance is evaluated by monitoring the pH variation of the marble’s surface covered by coatings in a controlled 1.2 × 1.2 cm area and a nitric acid solution with an initial pH of 4.0. Two kinds of marble (one purely composed of calcite and the other composed of mostly dolomite and minor calcite), coatings prepared DAP and DAP-IPA, and three enamel-like coatings are compared. The results in Figure 7 and Table 3 show that the acid resistance of these coatings is arranged in the following sequence: Enamel ≈ Enamel-CTAB > Enamel-DAP > DAP-IPA > marble (dolomite + calcite) > DAP > marble (calcite). In the first 30 min of soaking in the nitric acid solution, the pH variations mainly show the kinetic features. At 30 min, the pH of the solution containing the enamel-like coatings are 4.82–5.64, lower than the pH of the coating prepared by the two DAP methods (6.4 and 6.06). The pH after 60 min starts to stabilize and be controlled by thermal dynamic features. At 120 min, the pH of the solution containing the enamel-like coatings are 5.52–6.46, lower than the pH of the two DAP methods (7.62 and 6.93). The lower consumption of nitric acid suggests that the enamel-like apatite coatings displayed better acid resistance than the coatings prepared by DAP methods, which highlight the significance of phase and aggregation controls over the crystals within the protective coating. However, it is not expected that the coating prepared by the Enamel-DAP method is not as stable as the other enamel-like coatings. As all the three enamel-like apatite coatings have the sample compositions and phase and minor micro cracks still exist, the disadvantage of Enamel-DAP method may originate from surface etching by DAP in the pretreatment, which might activate calcite surface and make them more susceptible to acid in the micro cracks.

### 3.4. Mechanical Properties

**Figure 8:**
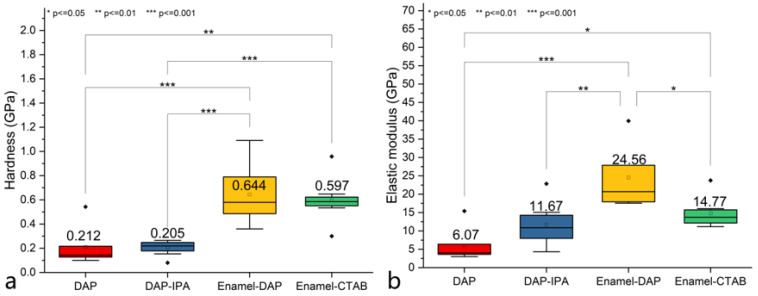
Hardness (**a**) and elastic modulus (**b**) of the enamel-like apatite coatings in comparison with coatings prepared by the DAP methods (outliers are marked as square dots).

The mechanical properties of enamel-like apatite coatings were evaluated by nano indentation in comparison with coatings prepared by the two DAP methods. As shown in Figure 8, the mean hardness of the enamel-like coatings is 0.644 GPa for the Enamel-DAP method and 0.597 GPa for the Enamel-CTAB method, which is approximate three times as high as those of the coatings prepared by the two DAP methods. Greater hardness generally leads to better abrasion performance, which provides the coating with better durability against potential wind erosion and scraping. This also results in reduced maintenance for conversed heritage structures. The mean elastic modulus of enamel-like apatite coatings is also higher than the coatings prepared by DAP methods. The Enamel-DAP coating is higher than the Enamel-CTAB coating in elastic modulus, which may have resulted from regional crystal orientations. Although both coatings prepared by Enamel-DAP and Enamel-CTAB methods exhibit better mechanical properties than those prepared by the DAP methods, they are still not comparable to natural enamel (hardness ~4 GPa) [38]. This is partially attributed to the absence of a bundle structure of nano rods in the prepared enamel-like coatings, which is characteristic of natural enamel. The acute and oblique nano rods shown in Figure 4 may also divert the indentation force.

## 4. Conclusions

Conserving outdoor marble relics requires specialized approaches that fully consider the stone’s unique characteristics and the environmental challenges it faces. The presented method manages to grow HAP nano rods perpendicular to the surface from the introduced HAP nuclei, which provides a new strategy to protect these valuable assets with enamel-like apatite coating. It is found that the solid content of HAP collosol should be controlled below 35 μg/cm^2^ to reduce microcracks in the coating. The hardness of this coating is around 0.6 GPa, approximately three times the value of phosphate coating prepared by the DAP method, which provides more durability against wind erosion, together with acid rain. This method does not erode the marble’s surface to obtain a calcium source from carbonates, as the diammonium phosphate or oxalate methods do, which would be more sustainable for ongoing maintenance. Even so, the potential of enamel-like apatite coating prepared on marbles has not been fully explored and more efforts are required to push the limits of its performance. For practical application on outdoor marble relics, the current solution-based method may find it difficult to scale up for large objects. A common measure in practice is to use pulp or cotton to maintain the solution for the growth of HAP, as the required growth time is only around 24 h or less. A hydrogel-based method would be more applicable and will be developed in the future.

## Figures and Tables

**Table 1:** Different preparation conditions and coating status of enamel-like coatings.

Entry	Pretreatment	HAP Collosol Applied (Solid Content, μg/cm^2^)	Additives in Mother Solution	Growth Time (h)	Coating Status
1	none	105.8	none	24	partially opaque and flaking
2	none	72.0	none	24	transparent but flaking
3	none	50.4	none	24	transparent, well attached, and with micro cracks
4	none	34.2	none	24	transparent, well attached, and micro cracks
5	none	10.2	none	24	transparent, well attached, and with micro cracks
6	none	0	none	24	No coating
7	none	35 *	ethanol	24	transparent, well attached, and with micro cracks
8	none	10.3	CTAB	24	transparent, well attached, and with minor micro cracks
9	1 mol/L DAP for 1 h	10.6	none	24	transparent, well attached, and with minimal micro cracks
10	1 mol/L DAP for 1 h	0	none	24	No coating
11	1 mol/L DAP for 1 h	35 *	none	2	transparent, well attached, and with minor micro cracks
12	1 mol/L DAP for 1 h	35 *	none	4	transparent, well attached, and with minor micro cracks
13	1 mol/L DAP for 1 h	35 *	none	8	transparent, well attached, and with minor micro cracks
14	1 mol/L DAP for 1 h	35*	none	12	transparent, well attached, and with minor micro cracks
15	1 mol/L DAP for 1 h	35 *	none	24	transparent, well attached, and with minor micro cracks
16	1 mol/L DAP for 1 h	35 *	none	48	transparent, well attached, and with minor micro cracks

Note: * the amount of HAP collosol was not precisely documented but was conducted as the standard of 35 ± 1 μg/cm^3^.

**Table 2:** Chemical composition of the enamel-like apatite coatings.

Preparation Method	Ca (at. %)	O (at. %)	P (at. %)	C (at. %)	F (at. %)	Na (at. %)	Mg (at. %)	Ca/P	P/F
Enamel	23.25 ± 0.33	52.99 ± 1.51	13.25 ± 0.58	6.38 ± 1.69	3.38 ± 0.33	0.69 ± 0.60	0.05 ± 0.05	1.75	3.92
Enamel-CTAB	23.90 ± 0.38	53.11 ± 1.58	13.43 ± 0.83	5.52 ± 2.28	3.53 ± 0.17	0.46 ± 0.40	0.04 ± 0.04	1.78	3.80
Enamel-DAP	23.19 ± 0.27	54.24 ± 0.69	12.99 ± 0.74	5.56 ± 0.80	3.39 ± 0.15	0.56 ± 0.49	0.08 ± 0.08	1.79	3.83

**Table 3:** pH of the solution containing the substrates or coatings after 30, 60 and 120 min.

Sample Label	pH		
	30 min	60 min	120 min
Marble (calcite)	6.80	8.10	9.24
Marble (dolomite + calcite)	6.05	6.67	7.22
DAP	6.40	7.08	7.62
DAP-IPA	6.06	6.47	6.93
Enamel	4.82	5.39	5.52
Enamel-CTAB	4.97	5.31	5.69
Enamel-DAP	5.64	6.16	6.46

## Data Availability

The original contributions presented in this study are included in the article. Further inquiries can be directed to the corresponding author.
